# A Narrative Review of Dietary Assessment Tools for Preschool-Aged Children in the Home Environment

**DOI:** 10.3390/nu14224793

**Published:** 2022-11-12

**Authors:** Laura L. Bellows, Yuanying Lou, Rachel Nelson, Ligia I. Reyes, Renae C. Brown, Noereem Z. Mena, Richard E. Boles

**Affiliations:** 1 Division of Nutritional Sciences, Cornell University, Ithaca, NY 14853, USA; 2 Department of Agriculture, Nutrition and Food Systems, University of New Hampshire, Durham, NH 03842, USA; 3 Anschutz Medical Campus, University of Colorado, Aurora, CO 80045, USA

**Keywords:** diet assessment, measurement, dietary intake, nutrition, home environment, preschool, early childhood, intervention, obesity prevention

## Abstract

Preschool-aged children in the U.S. have suboptimal diets. Interventions to improve child nutrition focus on parents and their role in shaping social and physical home environments, which influence children’s eating behaviors. Dietary assessment tools selected to measure intervention objectives, and how results are interpreted in key findings, are essential when examining children’s diets. The objectives of this review were to (1) describe dietary assessment tools used in intervention studies in young children focused within the home environment; and (2) examine how the application of these dietary assessment tools addressed intervention objectives. PubMed and Web of Science were searched for English-language nutrition intervention studies that included children aged 2–5 years, had a home environment component, used a dietary assessment tool, and reported on diet-related outcomes. Seventeen studies were included. Intervention objectives focused on overall diet, specific food groups, eating occasions, and obesity prevention/treatment. Concordance of key findings with intervention objectives, type of tool used, and multiple tools within the same study varied with 8 studies aligning in objective and tool, 1 discordant in both, and 8 partially concordant or too broad to determine. This review highlights current challenges in measuring dietary intake in preschoolers and provides recommendations for alternative applications and strategies.

## 1. Introduction

Early childhood is a period for growth and development and when eating habits and dietary behaviors are formed. Nutrition is a critical contributor to overall health and plays a vital role in the prevention of diet-related chronic diseases, such as obesity [1]. Unfortunately, young children in the United States (U.S.) have poor diet quality [2] and they continue to fall short in achieving adequate intake of nutritious foods, particularly vegetables, whole grains, and dairy [3]. Conversely, children have high consumption of added sugars, sodium and saturated fat when compared to the recommendations of the Dietary Guidelines for Americans [3]. U.S. children aged 2- to 5 years consume approximately 75 percent of daily energy intake at home, emphasizing the important role parents and the home food environment have on influencing children’s dietary intake [4,5]. The home is the child’s first food and eating environment, is fundamental in shaping the emergence of eating habits in early childhood and continues to be a critical environment throughout childhood [5].

Interventions targeting dietary intake and eating behaviors of preschool-aged children are increasingly focused on parents as agents of change, largely due to the extensive evidence supporting parents’ influence on shaping children’s eating and growth [6,7,8]. In families with young children, parents are widely considered the gatekeepers of the home environment [9], and as such, substantially contribute to the physical and social aspects of the home that may impact child dietary intake as well as weight status. The physical home food environment is influenced by food availability (physically in the home), accessibility (within reach by hand of the child), and purchasing behaviors (frequency of acquisition, socioeconomic position, and taste preferences) [10,11]. Socially, children’s eating behaviors are influenced by parent feeding styles and practices [12], expectations for diet [13,14], role modeling [13], and mealtime routines [6]. A growing body of evidence demonstrates the influence of the physical and social environments on dietary intake in children [15]. Accurate dietary assessment measures are needed to describe children’s diet, demonstrate dietary improvements via nutrition interventions, and better understand how the home environment influences dietary intake [16].

The degree of accuracy that is required to assess young children’s diets depends on the context and whether the selected tool fits the study’s purpose and can answer the stated research question(s) [17]. With the multidisciplinary nature of obesity research, it can be challenging for researchers to select the best dietary assessment method when designing studies and to appropriately interpret results [18]. Thus, it is imperative for researchers to understand the nuances between different diet assessment tools, including the dietary outputs produced by the tool and key considerations of use with young children (Table 1).

Approaches to measure dietary intake in the home environment are primarily self-report methods in which parents/caregivers report on the foods and beverages consumed by their child. These self-report measures include 24 h recall, food records, food frequency questionnaires (FFQ) and screeners, and food checklists. Objective measures, such as food photography and biomarkers (e.g., Veggie Meter), are minimally used in the home setting; however, as technologies continue to emerge, these methods are becoming more feasible [16,17,18,19].

Dietary assessment tools can be used to capture habitual or acute dietary intake as well as a variety of dietary outputs—foods and food groups consumed (e.g., fruits and vegetables), energy (calories), macronutrients (carbohydrates, fats and protein), micronutrients (vitamins and minerals), and dietary scores or quality indices (e.g., diet diversity, Healthy Eating Index) [18]. Intervention studies may use these dietary outputs to assess changes in overall diet composition, consumption of specific foods/food groups, intake by eating occasion, or the contribution of nutrients to health outcomes, including obesity. The ability to assess the efficacy and effectiveness of nutrition interventions to produce dietary changes is reliant on the use of psychometrically sound diet assessment tools to capture dietary variables that are in concordance with the intervention design and study objectives.

Key considerations for measuring young children’s diets include the use of a proxy respondent (parent, caregiver), the setting of intake (home, childcare), respondents’ ability to accurately capture child’s diet in a prescribed timeframe (24 h, eating occasion), respondent characteristics (education, literacy and culture) and respondent burden. Given the developmental stage of young children, an adult respondent is the primary source for preschoolers’ diet information. A large proportion of children aged 2–5 years split their days between childcare and home settings [19], limiting the ability of some adult respondents to accurately report on a child’s diet over a 24 h period, thus raising concerns of recall and reporting bias.

**Table 1:** Diet assessment tools used with preschool-aged children [18,20,21,22,23,24].

Dietary Assessment Tool	Description	Key Considerations for Use in Preschool-Aged Children	Respondent		Diet Intake Captured		Dietary Variable Outputs				
			Single	Multiple	Habitual	Acute	Foods/ Food Groups	Energy	Macro-Nutrients	Micro- Nutrients	Score/Index
24 h Recall	Facilitated interview by trained professional or automated software to capture amounts of foods and beverages consumed by respondent in past 24 h period. *Sample period:* 2–3 days, mix of weekday and weekend	Primary respondent may not be with child for all 24 h. Culturally specific foods can be captured due to open-ended nature of tool.	✓			✓	✓	✓	✓	✓	✓
Food Record/ Diary	Written or electronic account of all foods and beverages consumed over a specified timeframe. Items may be weighed or non-weighed. *Sample period:* 3–7 consecutive days	Primary respondent may not be with child for all 24 h. Record may be completed across multiple settings (childcare/home, split households). Culturally specific foods can be captured due to open-ended nature of tool.	✓	✓		✓	✓	✓	✓	✓	✓
FFQ/ FFQ Screener	Defined list of foods and beverages; asks frequency of consumption over an extended timeframe. Respondents choose from close-ended, multiple-choice options. Usually self-administered but can be interviewer administered/assisted. FFQ contains a comprehensive list of items (~120–180 items). FFQ screeners contain an abbreviated list of the specified items (~20 items) and can be targeted to a specific food group or nutrient. *Sample period:* 1 W to 6 M (vs. 12 M for adults)	Complex to navigate and literacy level of respondent should be considered. The FFQ Screener provides only high-level view of intake. List of food items may not include foods commonly consumed in some cultures or child-friendly items.	✓		✓		✓	*	✓	✓	✓
Food Checklist	Defined list of foods and beverages for which respondents are asked to check which of the specified items were consumed over a specified time period. It may also ask about behavioral habits (e.g., reading nutrition labels). Portion sizes may be captured. *Sample period*: single or multiple days	Low participant burden, although literacy level of respondent should be considered. List of food items may not include foods commonly consumed in some cultures or child-friendly items.	✓		✓	✓	✓				✓

Energy: calories/kilojoules; Macronutrients: proteins, carbohydrates, fats; Micronutrients: vitamins and minerals; Score/Index: diet diversity, Healthy Eating Index, etc. * Provides energy as an output but less accurate than other tools.

While previous reviews have examined diet assessment tools for children [25,26,27] few have included preschool-aged children, have restricted inclusion of assessments to short forms (i.e., ≤50 items) [28] or have narrowly focused on interventions aimed at the physical home food environment without social characteristics. Thus, the objectives of this review were to: (1) describe the dietary assessment tools used in intervention studies in young children focused within the home environment; and (2) examine how the application of these dietary assessment tools addresses intervention objectives.

## 2. Materials and Methods

### 2.1. Data Sources and Searching Strategies

The protocol of this review was developed based on the current recommendations set by the Preferred Reporting Items for Systematic Reviews and Meta-analysis Approach (PRISMA). Two electronic databases, PubMed and Web of Science, were searched in February 2022. The searching strategy was developed using a combination of Medical Subject Headings (MeSH) terms and key words relating to young children, home-environment interventions, diet assessment tools, intake and study type. University librarians, with expertise in systematic reviews, provided guidance on search terms to optimize the search strategy created for PubMed and adapt it for use in Web of Science. A time filter was used in both searches to include articles published only after 1 January 2010, which was selected to capture the most recent evidence and minimize overlap with studies included in prior reviews. The full list of searching terms is in Appendix A. In addition to the database search, manuscripts were identified via review of reference lists as well as studies citied within the selected manuscripts, particularly when a secondary analysis or methodology paper was initially identified.

### 2.2. Inclusion and Exclusion Criteria

To be included, manuscripts must have been written in English and published since 2010. Studies must have also included only healthy, typically developing preschool-aged children, 2–5 years, without known disorders or conditions that would have the potential to impact dietary intake (e.g., severe allergy or cystic fibrosis). Included studies must have conducted a nutrition intervention with a component focused on the home environment, including both the physical (e.g., food availability) and social (e.g., parent feeding practices) environments. Additionally, studies must have measured the child’s solid food and/or beverage intake (not infant formulas or breastmilk) in the home environment using a diet assessment tool and reported on a diet related outcome(s).

Secondary analysis, methodology manuscripts, review papers, cross-sectional studies, and validation studies were excluded from this review. Additionally, studies were excluded if the majority (>50%) of children fell outside 2- to 5-year age range at baseline, or if the study population was primarily on the upper or lower ends of the age range (e.g., 5–7 years old or 0–2 years old).

### 2.3. Selection of Studies

Following the search of databases, the identified manuscripts were imported to systematic review software, COVIDence (Veritas Health Innovation, Melbourne, Australia). After the removal of duplicates, title and abstracts were screened independently by two reviewers to identify eligible studies according to the inclusion and exclusion criteria. Full text articles of manuscripts deemed eligible were then imported to COVIDence for full-text review. The two reviewers independently conducted a full text review by including manuscripts that met inclusion criteria, then eliminated and recorded the reason(s) for each excluded manuscript. Any conflicts during the title and abstract review or full-text review were resolved through discussion between reviewers, and if needed, the additional consultation of a third reviewer. After completing the full-text review, included manuscripts that were written about the same intervention program were identified, and the most relevant manuscript was selected. This way, each intervention, rather than each manuscript, was the unit of interest for the review. For this review, manuscripts reporting study outcomes were prioritized.

### 2.4. Data Extraction

A template was created in Excel to extract study characteristics and outcome data. Data extraction was split and completed by two reviewers independently. For quality assurance, a subsample of included articles (20%) was completed by the two reviewers together. The following information was extracted: bibliographic information (first author’s last name, year of publication, and country of study), study population (children’s age, sample size, adult respondents’ role/gender, race, education level, and income level), intervention-related (intervention name, objective, target, length, and diet-related components in the intervention), and diet assessment-related (diet assessment tool(s), description of the tool(s) used, outcome variables, and key findings).

## 3. Results

**Figure 1:**
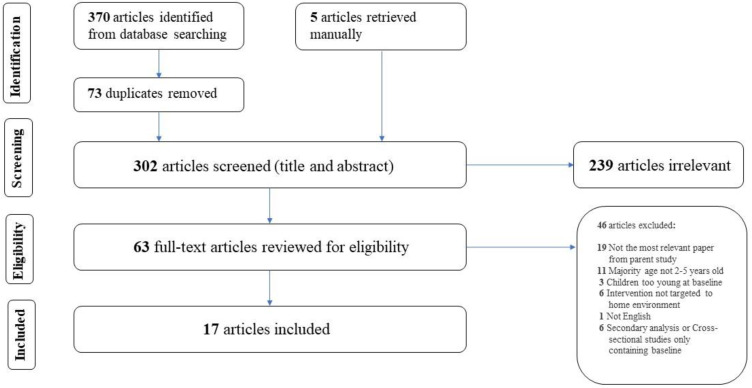
PRISMA flow chart [29] detailing identification, screening, and inclusion of articles.

A PRISMA flow chart that summarizes the results of the search, including reasons for exclusion is shown in Figure 1. The database searches initially identified 370 articles. After duplicates were removed, 297 articles were moved into titles and abstracts screening, and 58 were accessed and reviewed in full text for eligibility. During full text review, 5 manuscripts outside the initial database searches were identified from the references and subsequently imported manually for review. A total of 17 studies were included in this review.

### 3.1. Study Characteristics

Of the 17 studies reviewed, seven were located in North America (United States [30,31,32,33,34,35], Canada [36]), four in Australia [37,38,39,40], four in Europe (Finland [41], Sweden [42], Turkey [43], United Kingdom [44]), one in Asia (Sri Lanka) [45], and one in South America (Ecuador) [46]. Within the 16 studies that reported sample size, samples ranged from 30 [30] to 1211 [35]. In study design, nine were randomized controlled trials (RCTs) [31,33,35,37,38,40,41,42,45], five were pilots [30,34,36,39,44], and three were quasi-experimental [32,43,46]. Table 2 presents the results in detail.

### 3.2. Participant Characteristics

All studies included children within the 2- to 5-year age range, however, two studies did not report specific mean ages of child participants [45,46]. Female caregivers served as the majority (51–100%) respondent in fourteen studies [30,31,32,33,34,35,37,38,39,40,43,44,45,46], and the remaining three studies listed the respondent as parents of unspecified sex/gender [36,41,42]. Five respondent groups were low income [31,32,33,34,35], four were of mixed incomes [30,36,40,44], and the remaining eight did not report income status [38,39,41,42,43,46] or were unclear [37,45]. Of the 17 studies, only 11 reported details for race and ethnicity—five included majority White (48–94%) [30,32,34,36,44], two majority Hispanic (56–91%) [31,35], one majority Black (91%) [33], one majority Sinhalese (76%) and two that did not report majority but noted Indigenous individuals comprised 2% of their sample [38,40].

### 3.3. Measurement Tools Used

Nine of the 17 studies used an FFQ with 8 using a full FFQ [37,38,39,40,41,42,44,46] and 1 using an FFQ Screener [34]. Four studies used a Food Record [36,39,43,44], and of the remaining studies, 3 used a 24 h Recall [31,32,33] and 3 used a Food Checklist [30,35,45]. Three studies used multiple diet measures, 2 of which included objective measures (food photography and Veggie Meter [30], or weighed meal [39]) to complement the self-report dietary measures.

The sampling period varied across the different tools. Of the studies that used an FFQ or FFQ Screener, 6 studies had a sampling period that ranged from the previous 1 day to 6 months [25,28,29,31,32,37], and 3 studies did not report the sampling period [39,42,44]. For studies that used a Food Record, the sampling period was 3 days in 3 studies [36,39,43] and 4 days in 1 study [44]. All 4 studies included 1 weekend day. Studies that used a 24 h Recall had a sampling period of 2 days in 1 study [32] and 3 days in 2 studies [31,33], and all included 1 weekend day. Of the 3 studies that used a Food Checklist, the sampling period for 1 study was for the previous 1 week [45], and the remaining 2 studies did not report the sampling period [30,35].

Studies reported between 1 to 5 dietary variables. Among studies that used an FFQ or FFQ Screener, all 9 reported on the variable foods/food groups [34,37,38,39,40,41,42,44,46], with 6 reporting that as the only output variable [34,39,40,41,42,44]. One FFQ study also examined diet diversity [46] with the remaining 2 studies also reporting on energy and other nutrients [37,38]. Among studies using a Food Record, all 4 reported foods/food groups [36,39,43,44], 3 also reported energy and macronutrients [36,43,44], and 2 further reported micronutrients [36,43]. All 3 studies using a 24 h Recall reported on energy and macronutrients [31,32,33], and 1 additionally reported on foods/food groups [32]. Among those using a Food Checklist, all 3 reported foods/food groups [30,35,45], with one also reporting dietary diversity [45].

The psychometric properties of the tools used had limited reporting. Only 1 study reported on the psychometric properties of the tool used (FFQ) with citation of the original source [40]. Nine studies cited the original source for a given tool used [30,32,34,35,37,38,39,41,42], with 3 citing sources which were with populations other than preschool-aged children [30,34,39]. Nine studies did not fully report psychometric properties [30,31,33,36,39,43,44,45,46].

### 3.4. Intervention Scope

Interventions ranged in length from 3 weeks [44] to 3 years [31] and focused on a variety of topics related to the physical and social home food environment. Eleven interventions focused primarily on the social home food environment and addressed topics related to feeding practices, behavioral capability (knowledge and skills), self-efficacy, and role modeling [31,32,33,34,35,37,38,42,43,45,46]. Two studies focused on these social topics as well as the physical home food environment by addressing availability and accessibility of healthy or unhealthy foods with parents [40,41]. Two studies focused primarily on the physical home food environment by making target foods available to children [39,44]. Two studies provided limited information on intervention content [30,36].

### 3.5. Intervention Objectives

Diet-related intervention objectives, as described in the studies, were categorized into 4 groupings: 7 studies focused on overall diet [35,37,38,41,43,45,46], 4 on specific food groups [30,33,39,40], 1 study on eating occasions [44], and 5 studies on obesity prevention or treatment [31,32,34,36,42]. Among the studies grouped as overall diet, the primary objectives explicitly listed a dietary objective yet varied in terminology—dietary diversity [45], dietary intake [37], dietary patterns [38], food consumption [41], nutritional status [43], and nutrition [35,46]. Studies on specific food groups had objectives centered on fruits and/or vegetables [30,39,40], solid fats and added sugars (SoFAS) [33], or junk foods [35]. The only eating occasion study had objectives on snacks [44]. Among the 5 studies grouped in the obesity prevention and treatment category, all studies had broad objectives and only 2 listed diet or nutrition in the objectives [32,36]. None of the 5 studies listed specific diet-related study objectives yet all reported findings related to diet outcomes. Table 3 presents intervention objectives, diet assessment measures and key findings for each study.

### 3.6. Key Findings

Each study was reviewed to assess concordance of (1) diet-related intervention objectives with key findings, (2) key findings with the diet assessment tool used, and (3) key findings produced from multiple tools within individual studies. Eight of the 17 studies reported key findings that aligned with their objective and assessment tool(s) [30,33,36,39,40,41,44,45]. Key findings from 1 study were discordant with both the intervention objective and tool used, where its objective was on dietary patterns, its findings were on energy, and its assessment tool was an FFQ, which is suboptimal for measuring energy intake [38]. Three studies were partially aligned, where 1 matched in objectives and findings but used an FFQ to capture energy [37]; 1 inquired on nutrition and reported on dietary diversity but used an FFQ without baseline data [46]; and 1 inquired on nutritional status but reported on consumption patterns despite using an adequate tool (i.e., Food Record) for its key findings [43].

Five of the studies had objectives that were broad or did not specify diet in their objective yet reported on specific dietary outcomes [31,32,34,35,42]. Among those that were broad, 1 study broadly inquired about nutrition and reported specifically on fruit, vegetable, and junk food consumption [35]; another inquired about food parenting and weight outcomes and reported broadly on macronutrients and energy [32]. Those that did not specify diet in their objectives included one that inquired about Body Mass Index and reported on energy [31], another that inquired about healthy behaviors and reported on fruit and vegetable intake [34], and a third that inquired about obesity treatment and reported on sweet consumption [42]. Four of the five studies with objectives that were broad or did not specify diet-related outcomes were in the obesity prevention and treatment grouping [31,32,34,42].

Three of the 17 studies used multiple dietary assessment tools, 2 using a mix of self-report and objective measures to answer the same question [30,39] and 1 using multiple self-report measures to answer different research questions [44]. Bakirci-Taylor et al. (2019) [30] used 3 different tools: 1 self-report (Food Checklist) and 2 objective measures (Food Photography and Veggie Meter). For intervention effects, findings were discordant between data from self-report and objective tools, where the self-report showed a positive intervention effect and objective showed partial or no effect. Poelman et al. (2019) [39] used 3 different tools, 2 self-report (FFQ and Food Record) and 1 objective (Weighed Dinner Meal). For intervention effect, findings were discordant as FFQ showed partial positive intervention effect, Food Record showed no effect, and Weighed Dinner Meal showed no effect. Reale et al. (2018) [44] used 2 self-report tools (FFQ and Food Record). For intervention effect, differences were detected between snack control strategies at increasing vegetable intake (Food Record) and no changes in frequency of snack intake was detected (FFQ).

## 4. Discussion

This review summarized the application of dietary assessment tools used in intervention studies focused on young children in the home environment. Of the 17 studies examined, 12 studies used fixed question sets to examine dietary intake over a designated time frame (9 FFQ [34,37,38,39,40,41,42,44,46] and 3 food checklists [30,35,45]) and 7 used open-ended assessments over multiple 24 h periods (3, 24 h recalls [31,32,33] and 4 food records [36,39,43,44]). Only 6 studies reported on the psychometric properties of tools that were tested with preschool audiences [32,35,37,38,41,42]. The studies in this review had intervention objectives that were grouped into 4 categories: overall diet, foods/food groups, eating occasion, and obesity prevention and treatment. Intervention objectives varied in specificity and the use of nutrition terminology. Only 8 of the studies’ intervention objectives fully aligned with their study findings [30,33,36,39,40,41,44,45]. An additional 3 studies [37,43,46] partially aligned and 1 study was discordant [38]. Five studies, four of which were in the obesity prevention and treatment grouping, were broad or did not have a specific dietary objective [31,32,34,35,42]. Parents and caregivers served as proxy respondents in all studies, with 2 studies collecting complementary data directly with children through objective measures—weighed dinner [39] and Veggie Meter [30].

Findings suggest that dietary assessment tools used with preschool-aged children are most often applied to answer a variety of research questions with no agreement on standards for assessment, including which foods to measure, the time frame to consider, and related social characteristics impacting the home food environment. Further, some but not all intervention objectives matched reported key findings. Collectively, these findings point to challenges in obtaining dietary data on preschool-aged children, particularly with the reliance on parents and caregivers as proxy respondents, as well in interpretation of the data obtained from the self-report dietary measures.

To improve children’s diet, it is necessary to accurately measure their current intake. The accuracy is reliant on validity of the tool itself as well as reliability of the respondents’ input. Reporting the dietary intakes of young children, particularly in the context of obesity, brings with it additional challenges and considerations. These include the need for a proxy respondent (e.g., parent or caregiver), consideration of developmental stage (e.g., cognitive skills), and food consumption away from home [18]. All studies in this review used parents or caregivers as respondents, with 7 studies using a 24 h recall or daily food records [31,32,33,36,39,43,44]. The likelihood of recall and reporting bias are high for proxy respondents, particularly if they are asked to report on 24 h intake in which they are not present for some of the eating occasions or if multiple respondents split the reporting over the 24 h period [47]. Thus, researchers should consider if a full day’s intake is necessary versus examining eating occasions in which the caregiver is with the child (e.g., dinner) and/or those time intervals in which the intervention is specifically targeting. If the intervention target is on parents serving their children more fruits and vegetables, then collecting dietary data when the child is outside of parental care may not be reliable from a measurement perspective, nor produce a valid intervention effect. Lastly, respondent characteristics such as educational level, reading and digital literacy, and inclusion of food relevant to respondents’ culture (e.g., ethnic background, geographical location) should be considered for accuracy along with respondent burden.

The accuracy of dietary data relies on the use of psychometrically sound tools that are critically assessed with the sample population. The reporting of psychometric properties for the studies included in this review were limited in detail and scope. Nine studies in this review did not fully report psychometric properties of reliability and validity [30,32,34,35,36,37,38,39,41,42] and an additional 3 studies reported sources in which testing was done on populations other than preschool-aged children [30,34,39]. This illustrates not only the need for better measures for early childhood audiences, but also more awareness and/or training amongst researchers in dietary measurement. This is critical as the use of methods with low validity greatly attenuates the associations between dietary intakes and outcomes in health [16].

Due to the inherent limitations of self-report diet assessment tools, combined with challenges of working with early childhood audiences and sparse psychometrics, considerations for using multiple methods to maximize the strengths of each instrument may be warranted. The use of alternative approaches to capture diet as well as interpret outputs from dietary measures may provide researchers the ability to answer more clearly defined research questions to strengthen the concordance of intervention objectives and study design. Two studies in this review coupled self-report dietary data with objectives measures, including food photography and biomarkers (e.g., Veggie Meter) [30] and a weighed dinner meal [39] to evaluate different aspects of their intervention. A third study used multiple dietary tools to examine different aspects of their intervention related to an eating occasion, snacking [44].

As technology becomes more ubiquitous with daily life, utilizing digital devices to capture dietary information in young children has potential. Food photography is an emerging tool for dietary assessment as an image-assisted method to enhance another dietary assessment method, as used in the Bakirci-Taylor et al. (2019) study [30], or as the primary form of dietary data [48]. Food photography has been used with preschool audiences to assess a 24 h period [49] or a single eating occasion, such as lunch [50] and dinner [51,52]. In addition to providing energy and nutrients [52], data produced by food photography has been used to examine dietary quality via the Healthy Meal Index [51,53] and comparison to dietary recommendations, namely the Child and Adult Care Food Program’s nutritional standards [50,52]. Lastly, McCloskey et al. (2019) [51] used food photography to examine the context of foods served to preschoolers in the home environment by examining concordance with mothers’ foods served and timing of meal, as well as food preparation (e.g., takeout to non-convenience). Food photography provides an alternative method to collecting dietary data for preschool audiences as well as innovative approaches to interpreting these data.

In addition to alternative methods, researchers should consider alternative approaches to capturing food or diet-related data beyond just intake. When examining studies in this review, only 2 studies [40,41] addressed food availability and accessibility in their interventions, with an additional 2 studies [39,44] making target foods available to their participants. Both cross-sectional and longitudinal studies demonstrate that home food availability can predict child food intake [10,54,55]. For instance, a study by Boles et al. (2019) [10] examined the relationship between the HFE and child dietary intake of preschool-aged children from rural and low-income, culturally diverse families and found that the availability of fruits and vegetables, meat products and sugar sweetened beverages were shown to predict greater intake by preschool children [10]. Because food availability has been shown to be predictive of dietary intake, researchers may consider using measurement tools that capture the availability of foods in the home as an alternative or complement to dietary intake measures.

There are several limitations to this review. First, it only included peer-reviewed full-text, English publications, hence publications in languages other than English or in the gray literature may exist and provide additional insights on this topic. Next, we only included 2 databases in our review. Collectively, these limitations may have influenced the authors’ ability to find and interpret the literature on this topic. This narrative review has several strengths, including using the most updated guidelines on completion of systematic reviews (PRISMA). Two reviewers screened full articles for inclusion and extraction with a high level of agreement. To note, while neither a strength or limitation, most of the intervention studies included in this review focused on childhood obesity and reported diet as a primary outcome. This is likely due to the high global prevalence, resulting in increased funding for such studies during the time period of our search (2010–2022).

## 5. Conclusions

This review found variation in the concordance of key findings with intervention objectives, type of dietary assessment tool used, and multiple tools within the same study. The use of traditional, self-report dietary assessment measures with preschool audiences to capture accurate data is challenging. The need for proxy respondents, coupled with limited psychometric properties of existing tools, calls for more methodological work as well as innovative approaches to capture and interpret young children’s dietary data. Approaches to advance objective measures, such as food photography, and the expansion of research questions beyond just daily intake are warranted. This could include examining food availability in the home environment or capturing intake by eating occasions instead of over a full day in which the child is in various settings (e.g., preschool, home) with multiple respondents (e.g., parent, teacher, another caregiver). These types of approaches could improve the concordance between intervention objectives, dietary assessment tools, and key findings, and ultimately provide a more robust understanding of young children’s diets and the efficacy of nutrition interventions.

## Figures and Tables

**Table 2:** Intervention studies and dietary assessment tools used to measure young children’s diet in the home environment (*n* = 17).

Study	Design	Child Participant		Adult Respondent			Dietary Assessment Tool			Dietary Variables Reported	Psychometric Properties
		Sample Size	Age (y) Mean (SD)	Role Gender	Race/ Ethnicity	Income	Type	# Items	Sample Period		
Wyse et al. (2012), Australia [40]	RCT	*n* = 394	4.3 (0.6)	Parents 96% female	Indigenous (2%)	Mixed	FFQ	NR	Previous 1 d, 7 d	foods/food groups (F/V)	Tool psychometrics reportedOriginal source cited (pre-K)
Davison et al. (2013), USA [32]	Quasi-Exp	*n* = 423	3.6 (1.0)	Parents/Grandparents 92% female	White (68%) Black (22%)	Low	24 h Recall	-	2 d(1 Wd, 1 We)	energy macronutrients foods/food groups (multiple)	Original source cited (pre-K)
Duncanson et al. (2013), Australia [38]	RCT	*n* = 146	4.0 (0.1) I 4.0 (0.9) C	Parents 99% female	Indigenous (2%)	NR	FFQ	120	Previous 6 M	energy macronutrients foods/food groups (multiple)	Original source cited (pre-K)
Natale et al. (2014), USA [35]	RCT	*n* = 1211	3.9 (0.9)	Caregivers 90% female	Hispanic (56%) Black (33%)	Low	Food Checklis	32	NR	foods/food groups (F/V, junk food)	Original source cited (pre-K)
Roche et al. (2017), Ecuador [46]	Quasi-Exp	NR	NR	Mothers	Indigenous (% NR)	NR	FFQ	NR	Previous 2 W	foods/food groups (multiple) dietary diversity	NR
Barkin et al. (2018), USA [31]	RCT	*n* = 610	4.3 (0.9)	Parents 98% female	Hispanic (91%)	Low	24 h Recall	-	3 d(2 Wd, 1 We)	energy macronutrients	NR
Ling et al. (2018), USA [34]	Pilot	*n* = 69	4.5 (0.5)	Caregivers 96% female	White (48%) Black (39%)	Low	FFQ Screener	41	Previous 1 W	foods/food groups (multiple)	Original source cited (other)Sample specific data provided
Mirotta et al. (2018), Canada [36]	Pilot	*n* = 45	3.2 (0.2)	Parents	White (81%)	Mixed	Food Record(non-weighed)	-	3 d(2 Wd, 1 We)	energy macronutrients micronutrients foods/food groups (multiple)	NR
Reale et al. (2018), UK [44]	Pilot	*n* = 46	3.1 (0.8)	Mothers	White British, mixed or other (94%)	Mixed	FFQ	NR	NR	foods/food groups (F/V, snacks)	NR
							Food Record (weighed)	-	4 d(1 We)	energy macronutrients foods/food groups (F/V, snacks)	NR
Aktaç et al. (2019), Turkey [43]	Quasi-Exp	*n* = 74	5.5 (0.5) I-1 5.7 (0.2) I-2 5.1 (0.6) C	Parents 51% female	NR	NR	Food Record	-	3 d(2 Wd, 1 We)	energy macronutrients micronutrients foods/food groups (multiple)	NR
Bakirci-Taylor et al. (2019), USA [30]	Pilot	*n* = 30	3.6 (1.4) I 3.8 (0.8) C	Parents 100% female	White (73%) Hispanic (13%)	Mixed	Food Checklist	10	NR	foods/food groups (F/V)	Original source cited (pre-K)
							Food Photography	-	3 d(2 Wd, 1 We)	foods/food groups (F/V)	NR
							Veggie Meter	-	-	micronutrients	Original source cited (other)
Fisher et al. (2019), USA [33]	RCT	*n* = 119	3.7 (0.8)	Mothers (biological)	Black (91%)	Low	24 h Recall	-	3 d(2 Wd, 1 We)	energy macronutrients (SoFAS)	NR
Poelman et al. (2019), Australia [39]	Pilot	*n* = 32	5.2 (0.8) I-1 5.1 (0.7) I-2 5.0 (1.1) C	Parents 67% female	NR	NR	FFQ	NR	NR	foods/food groups (V)	Original source cited (other)
							Food Record (weighed)	-	3 d(2 Wd, 1 We)	foods/food groups (V)	NR
							Weighed Dinner Meal	-	1 meal	foods/food groups (V)	NR
Ray et al. (2020), Finland [41]	RCT	*n* = 802	5.2 (1.1) I 5.1 (1.0) C	Parents	NR	NR	FFQ	51	Previous 1 W	foods/food groups (F/V, sugary foods and beverages)	Original source cited (pre-K)
Somaraki et al. (2020), Sweden [42]	RCT	*n* = 174	5.2 (0.8)	Parents	NR	NR	FFQ	NR	NR	foods/food groups (multiple)	Original source cited (pre-K)Adaptions cite original source (other)
Ashton et al. (2021), Australia [37]	RCT	*n* = 125	3.9 (0.5)	Mothers	NR	Unclear	FFQ	120	Previous 2 M	energy macronutrients micronutrients foods/food groups (multiple) overall diet quality	Original source cited (pre-K)
Sirasa et al. (2021), Sri Lanka [45]	RCT	*n*= 345	NR	Parents/Caregivers 86% female	Sinhalese (76%) Muslim (21%)	Unclear	Food Checklist	NR	Previous 1 W	foods/food groups (multiple) dietary diversity	Checklist: NRScoring: Original source cited (pre-K)

C: control; d: day; FFQ: food frequency questionnaire; F = fruit(s); I: intervention; M: month(s); NR: not reported; pre-K: pre-kindergarten; Quasi-Exp: quasi-experimental; RCT: randomized controlled trial; SoFAS: solid fat and added sugar; V= vegetable(s); W: week(s); Wd: weekday; We: weekend day; y: years. Psychometrics: Notes indicate if the original source cited was conducted with pre-K (preschool-aged) or other (e.g., older children, adults) populations.

**Table 3:** Application of dietary assessment tools to address intervention study objectives related to young children’s diet (*n* = 17).

Study	Diet-Related Intervention Objective(s)	Intervention Duration	Assessment Tool(s) (Sample Period)	Data Collection Time Points	Key Findings
**Overall Diet**					
Aktaç et al. (2019), Turkey [43]	Evaluate the effectiveness of family participation in nutrition education on the nutritional status of preschool-aged children.	10 W	Food Record (3 d)	B, 10 W	The intervention led to positive changes in consumption patterns in the Family Participation Group (FPG) and Education Group (EG), with greater changes in FPG (e.g., energy percentage from proteins).
Ashton et al. (2021), Australia [37]	Evaluate the efficacy of a family-based lifestyle intervention on change in dietary intake in fathers and their preschool-aged children and; investigate associations in father–child dietary intakes.	8 W	FFQ (Previous 2 M)	B, 10 W, 9 M	Medium group-by-time effect sizes identified at 10 W for sodium and energy from core foods, energy-dense, nutrient-poor foods and prepacked snacks; sustained at 9 M follow-up. Moderate to strong associations existed in father–child dietary intakes for some dietary variables (e.g., fast foods).
Duncanson et al. (2013), Australia [38]	Determine if provision of quality nutrition information to rural parents using self-directed, technology-based education resources has an effect on the dietary patterns of 2–5-year-old children.	12 M	FFQ (Previous 6 M)	B, 3 M, 12 M	Total reported energy from nutrient-dense food groups and percentage energy from energy-dense, nutrient-poor foods were high at baseline relative to estimated total energy expenditure for child age. No intervention effect.
Natale et al. (2014), USA [35]	Assess the effectiveness of a child care-based parent and teacher healthy lifestyle role-modeling program on child nutrition and PA outcomes.	1 School Year	Food Checklist (NR)	B, Post-intervention	Intervention group significantly increased child FV consumption. Junk food consumption significantly decreased in the intervention group, increased in the control group.
Ray et al. (2020), Finland [41]	Evaluate the effects of a preschool-based family intervention on children’s energy balance-related behaviors such as food consumption and screen time, as well as PA and self-regulation skills.	5 M	FFQ (Previous 1 W)	B, 5 M	No significant differences were detected between intervention and control groups for energy balance-related behaviors, self-regulation skills, or for consumption frequencies of sugary everyday foods and beverages, sugary treats, and FV.
Roche et al. (2017), Ecuador [46]	Assess the nutritional, social, and cultural potential of mothers’ cooking clubs that promoted Quichua culture and foods (leafy greens) to improve children’s nutrition.	12 M	FFQ (Previous 2 W)	12 M	Mothers in the intervention group were ~10 times more likely to feed their children the leafy greens than controls. Dietary diversity scores for all promoted foods were significantly greater for intervention children than for control children.
Sirasa et al. (2021), Sri Lanka [45]	Evaluate the effectiveness of a multicomponent intervention of child nutrition education plus family engagement, compared to a single component and control, on children’s dietary diversity.	6 W	Food Checklist (Previous 1 W)	B, 6 W	Neither the Multicomponent nor Single Component groups showed significant differences in average dietary diversity score of children between baseline and post-intervention.
**Specific Food Groups**					
Bakirci-Taylor et al. (2019), USA [30]	Explore the effect of a parent-focused intervention with three mHealth technologies on the accessibility and intake of FV in young children.	10 W	Food Checklist (NR)	B, 10 W	Screener data showed intervention children had higher vegetable consumption than control. Food photos showed no significant effects for week x treatment or treatment of frequency of FV. Significant week x treatment interaction values in children’s Veggie Meter values were found in the intervention group compared to control at mid- and post-intervention.
			Food Photography (3 d)	B, 5 W, 10 W	
			Veggie Meter	B, 5 W, 10 W	
Fisher et al. (2019), USA [33]	Evaluate the efficacy of an authoritative food parenting intervention for low-income mothers to reduce preschool-aged children’s intake of calories from SoFAS.	12 W	24 h Recall (3 d)	B, 12 W	At post-intervention, children in the intervention group consumed ~ 94 kcal or 23% less daily energy from SoFAS than control group. Child total daily energy intake did not significantly differ between groups post-intervention.
Poelman et al. (2019), Australia [39]	Evaluate the effectiveness of repeated exposure to multiple vs. single target vegetables in increasing young children’s vegetable intake.	5 W	FFQ, (NR)	B, 4 M	FFQ data showed usual vegetable intake increased in the multiple target group from 0.6 to 1.2 servings/day and did not change in other groups. Food record data were not significant. Vegetable intake from the weighed dinner meal was not significantly different between groups.
			Food Record (3 d)	B, 5 W, 4 M	
			Weighed Dinner Meal	B, 5 W	
Wyse et al. (2012), Australia [40]	Assess the efficacy of a telephone-based intervention for parents to increase FV consumption in their 3–5-year-old children.	4 W	FFQ (Previous 1 d, 7 d)	B, 2 M, 6 M	FV scores were significantly higher in the intervention group than control group at 2 M and 6 M. Sensitivity analysis showed intervention effect at 2 M but not 6 M.
**Eating Occasion**					
Reale et al. (2018), UK [44]	Explore the preliminary efficacy of two strategies of snack portion control (reduction and replacement) and; examine the efficacy of these two methods to improve diet in preschoolers.	3 W	FFQ, (NR)	1 W, 7–9 W	Snack replacement resulted in increased vegetable intake and decreased total daily energy intake when compared to snack reduction (Food Record). No significant changes to the frequency of snack intake pre- and post-intervention (FFQ).
			Food Record (4 d)	1 W, 2 W, 3 W	
**Obesity Prevention and Treatment**					
Barkin et al. (2018), USA [31]	Test the effect of a multicomponent behavioral intervention on child BMI growth trajectories among preschool children at risk for obesity.	36 M	24 h Recall (3 d)	B, 12 M, 24 M, 36 M	The intervention resulted in a statistically significant reduction in mean child daily energy intake and higher percentage of energy from protein compared with control.
Davison et al. (2013), USA [32]	Test initial efficacy of a parent-centered, community-based participatory research approach for obesity prevention for improving food, PA, and media-related parenting, as well as child behavioral and weight outcomes.	6 M	24 h Recall (2 d)	B, 8 M	At post-intervention, children had significantly lower total energy intake and macronutrient intake (fat, protein, and carbohydrate) compared with pre-intervention.
Ling et al. (2018), USA [34]	Examine the feasibility and preliminary efficacy of using Facebook in a lifestyle intervention to improve healthy behaviors and reduce BMI.	10 W	FFQ Screener (Previous 1 W)	B, 10 W	The intervention resulted in small but nonsignificant effects on children’s FV intake.
Mirotta et al. (2018), Canada [36]	Examine the effectiveness of a home-based obesity prevention intervention on health behaviors, obesity risk and dietary intakes.	6 M	Food Record (3 d)	B, 6 M	Post-intervention, the 4 home visits (HV) group had significantly higher fiber intake. The 4 HV and 2 HV groups had significantly higher fruit intake compared to control.
Somaraki et al. (2020), Sweden [42]	Evaluate the effects of two approaches (a parent support program with and without booster sessions and standard treatment) to treat obesity in preschoolers.	12 M	FFQ (NR)	B, 3 M, 6 M, 12 M	Changes in intake did not differ between children by treatment. Within group changes in cookies/buns decreased over time among children in two treatment groups. Control children significantly decreased consumption of sweets/ chocolate.

B: baseline; BMI: body mass index; d: day; FFQ: food frequency questionnaire; FV: fruit(s) and vegetable(s); M: month; NR: not reported; PA: physical activity; SoFAS: solid fat and added sugar; W: week.

## Data Availability

No new data were created or analyzed in this study. Data sharing is not applicable to this article.

## Appendix A

### Appendix A.1. PubMed Search Terms

Child, Preschool[MeSH] OR “young child*”[tiab] OR kid[tiab] OR preschool*[tiab] OR toddler*[tiab]
“parents”[MeSH] OR “home environment”[MeSH] OR “home-based”[tiab] OR “home environment”[tiab] OR “home food environment”[tiab] OR “family meal”[tiab] OR “home food”[tiab] OR “food in home”[tiab] OR “family-based”[tiab] OR “family environment”[tiab] OR “family food environment”[tiab] OR caregiver[tiab] OR parent[tiab] OR famil*[tiab] OR mother[tiab] OR father[tiab] OR guardian[tiab]
AND
“diet recall*”[tiab] OR “food recall*”[tiab] OR “24 h recall*”[tiab] OR “food frequency questionnaire*”[tiab] OR FFQ[tiab] OR screener[tiab] OR “dietary questionnaire”[tiab] OR “food survey*”[tiab] OR “diet survey*”[tiab] OR “meal survey*”[tiab] OR “food record*”[tiab] OR “diet record*”[tiab] OR “food checklist*”[tiab] or “food behavior checklist*”[tiab] OR “diet checklist*”[tiab] OR “food photo*”[tiab] OR “photo assisted diet assessment*”[tiab] OR PADA[tiab] OR “image based food record*”[tiab] OR IBFR[tiab] OR “dietary observ*”[tiab] OR “meal observ*”[tiab] or “food observ*”[tiab] OR “diet assess*”[tiab] OR “food assess*”[tiab] OR “meal assess*”[tiab] OR “weighed record*”[tiab] OR “plate waste”[tiab] OR “direct weighing method*”[tiab] OR “food diar*”[tiab] OR “meal diar*”[tiab]
AND
“food intake”[tiab] OR “energy intake”[tiab] OR “nutrition intake”[tiab] OR “dietary intake”[tiab] OR consumption[tiab] OR “diet quality”[tiab] OR “diet variety”[tiab] OR “food group”[tiab] OR calorie[tiab] OR kcal[tiab] OR “energy density”[tiab] OR adequa*[tiab] OR “healthy eating”[tiab]
AND
Interven*[tiab] OR trial[tiab] OR RCT[tiab] OR “randomized control trial”[tiab] OR “randomized control trial”[tiab] OR “quasi experiment*”[tiab] OR “behavior change”[tiab]

### Appendix A.2. Web of Science Search Terms

TS = (“young child*” OR kid OR preschool* OR toddler*)
AND
TS = (“parent” OR “home environment” OR “home-based” OR “home food environment” OR “family meal” OR “home food” OR “food in home” OR “family-based” OR “family environment” OR “family food environment” OR caregiver OR parent OR famil* OR mother OR father OR guardian)
AND
TS = (“diet recall*” OR “food recall*” OR “24 h recall*” OR “food frequency questionnaire*” OR FFQ OR “screener” OR “dietary questionnaire” OR “food survey*” OR “diet survey*” OR “meal survey*” OR “food record*” OR “diet record*” OR “food checklist*” or “food behavior checklist*” OR “diet checklist*” OR “food photo*” OR “photo assisted diet assessment*” OR PADA OR “image based food record*” OR IBFR OR “dietary observ*” OR “meal observ*” or “food observ*” OR “diet assess*” OR “food assess*” OR “meal assess*” OR “weighed record*” OR “plate waste” OR “direct weighing method*” OR “food diar*” OR “meal diar*”)
AND
TS = (Interven* OR trial OR RCT OR “randomized control trial” OR “randomized control trial” OR “quasi experiment*” OR “behavior change”)
